# Transperineal Ultrasound Assessment of a Cystocele’s Impact on the Bladder Neck Mobility in Women with Stress Urinary Incontinence

**DOI:** 10.3390/medicina55090562

**Published:** 2019-09-03

**Authors:** Maria-Patricia Rada, Răzvan Ciortea, Andrei Mihai Măluțan, Doru Diculescu, Costin Berceanu, Mihaela Oancea, Cristian Ioan Iuhas, Carmen Elena Bucuri, Andrei Roman, Dan Mihu

**Affiliations:** 1 2nd Department of Obstetrics-Gynaecology, “Iuliu Hatieganu” University of Medicine and Pharmacy, 400012 Cluj-Napoca, Romania; mpr1388@gmail.com (M.-P.R.); malutan.andrei@gmail.com (A.M.M.); ddiculescu@yahoo.com (D.D.); mihaelaoancea321@yahoo.com (M.O.); iuhascristianioan@yahoo.co.uk (C.I.I.); cbucurie@yahoo.com (C.E.B.); dan_mihu@yahoo.com (D.M.); 2 Department of Obstetrics-Gynaecology, University of Medicine and Pharmacy Craiova, 200349 Craiova, Romania; dr_berceanu@yahoo.com; 3 Department of Radiology, The Oncology Institute Prof. Dr. “Ion Chiricuţă”, “Iuliu Hatieganu” University of Medicine and Pharmacy, 400012 Cluj-Napoca, Romania; andrei.roman678@gmail.com

**Keywords:** stress urinary incontinence, bladder neck mobility, transperineal ultrasound, cystocele, newborn weight

## Abstract

*Background and objectives*: As pelvic floor disorders are often difficult to assess thoroughly based on clinical examination alone, the use of imaging as a complementary technique is helpful. This study’s aim was to investigate by transperineal ultrasound (US) if there was any significant difference in the mobility of the bladder neck in women with stress urinary incontinence (SUI) without a cystocele and in those with SUI and an associated cystocele. The study also investigated whether the number of vaginal births and/or the heaviest newborn’s birth weight was correlated with the bladder neck mobility. *Materials and Methods*: A total of 71 women suffering from SUI were included in the study and divided into two groups based on the presence of a cystocele. Their bladder neck mobility was evaluated by transperineal US, calculating the distance from the inferior margin of the symphysis pubis to the bladder neck (SPBN), and the dorsocaudal linear movement (DLM), term used to illustrate the displacement of the bladder neck by subtracting rest and Valsalva values. GraphPad Prism 8 was used for statistical analysis. *Results*: Within both study groups, the SPBN values were significantly higher and the DLM values were significantly lower at rest as compared to Valsalva maneuver (*p* < 0.05). No significant difference between the groups regarding SPBN and DLM values at rest, Valsalva, or subtraction was demonstrated. A significant positive correlation was found between the bladder neck mobility and the heaviest newborn’s birth weight, regardless of the presence of a cystocele (*p* = 0.042). *Conclusions*: The presence of a cystocele had no significant impact on the bladder neck mobility measurements in patients with SUI. The heaviest newborn’s birth weight positively correlated with bladder neck hypermobility, as quantified by SPBN.

## 1. Introduction

The recent focus of research on the pathophysiology of stress urinary incontinence (SUI) has switched from the mid-urethra to the bladder neck, which is to some extent due to the therapeutic effect of different agents injected close to the bladder neck. SUI occurs when an intrinsic interaction between anatomical and functional factors is perturbed. This manuscript reveals findings related to the changes in the anatomical components of this mechanism.

Urethral hypermobility and intrinsic sphincter deficiency are the principal pathophysiologic mechanisms of stress urinary incontinence (SUI) [1], which is the most common type of urinary incontinence reported by women [2]. Bladder neck/urethral hypermobility, SUI, and cystocele often coexist, and their occurrence is usually triggered by common factors, among which childbirth perineal trauma is the most frequent [3,4].

Perineal trauma during childbirth leads to the damage or weakening of the pelvic muscles [5], and other perineal tissues and a cystocele may appear consequently. According to current knowledge, it is yet to be documented whether the presence of a cystocele influences bladder neck mobility. The hypothesis of our study was that there is no significant difference in the mobility of the bladder neck in women with SUI without a cystocele, and those with SUI and an associated cystocele. In addition, we tested another hypothesis, assuming that the number of vaginal births and/or the heaviest newborn’s weight are not correlated with the mobility of the bladder neck in patients with SUI, with or without a cystocele.

In the diagnosis of SUI, besides a thorough clinical examination, the evaluation of bladder neck mobility is performed using complementary diagnostic techniques such as the Q-tip test [6], cystourethrography [7], and imaging of the pelvic floor via transperineal ultrasound (US) [8,9,10].

Transperineal US may be used for morphological dynamic assessment of the bladder neck and urethra and allows reproducible quantitative measurements. Based on these premises, we used this technique that allowed us to perform specific measurements, and thus, to quantify the mobility of the bladder neck.

## 2. Materials and Methods

This study was conducted over a 9-month period in a University Hospital (‘Dominic Stanca’ Obstetrics-Gynecology Clinic) in Cluj-Napoca, Romania. All subjects gave their informed consent for inclusion before they participated in the study (see Supplementary Materials). The study was conducted in accordance with the Declaration of Helsinki, and the study protocol was approved by the Ethics Committee of the University of Medicine and Pharmacy “Iuliu Hațieganu” Cluj-Napoca (number 169/02.04.2017).

This was a prospective, observational analytic, case-control study. Study participants were women suffering from SUI, with or without an associated cystocele. The patients were divided into two groups: A control group composed of patients with SUI without cystocele (*n* = 33) and a case group composed of patients with SUI and cystocele (*n* = 38). A total of 71 women who had not previously undergone any surgical procedure for SUI or pelvic organ prolapse were included in the study, and consented to undergo transperineal US as part of their preoperative workup for SUI. The inclusion and exclusion criteria are presented in Table 1.

The Pelvic Organ Prolapse Quantification assessment tool (POP-Q) [11] was used to evaluate the patients with cystocele. Only patients with grade 2 or 3 prolapse of the anterior pelvic compartment prolapse were included. The POP-Q measurements were performed by the principal investigator of this study.

US measurements of the bladder and urethra parameters may yield different values based on the degree of bladder filling. Given that it was documented that the bladder is less mobile when it is full [8] and thus may prevent the complete development of the cystocele, for the purpose of this study, the measurements were performed when the urinary bladder was partially filled to ≈75 mL of urine.

Two-dimensional transperineal US datasets were obtained for analysis. A Toshiba Aplio US machine with a 3.5-MHz convex transducer was used to perform the examinations. All patients were examined at rest and on Valsalva maneuver, in dorsal lithotomy position, in a 30–45 min post voiding interval. Mid-sagittal images were obtained. The transducer, covered with a non-powder glove to avoid artifacts, was placed longitudinally to the vestibule and slightly tilted upwards, allowing scans of the anterior pelvic compartment [8,12]. In the dynamic phase, the increase in intra-abdominal pressure was achieved through Valsalva maneuver, and the measurements were obtained on a still image generated at the time of maximum displacement of the bladder neck by using the cine-loop technique [13].

The bladder neck was evaluated by studying its relationship with an anatomical landmark represented by the inferior margin of the pubic symphysis. The image acquisition and screen display were standardized, so that the transducer appeared at the top, and the left side was represented by the ventral aspect of the patient. Once the inferior edge of the symphysis pubis, the bladder, urethrovesical junction, and the urethra were visualized during rest, the image was frozen and placed on one side of the screen. Consequently, the participants were asked to perform the Valsalva maneuver and the new image was frozen and placed on the other half of the screen [14]. The position of the bladder neck was analyzed according to a reproducible method [13] using an XY-coordinate system. The *X*-axis was a vertical line tangent to the inferior margin of the symphysis pubis, and the *Y*-axis was perpendicular to the *X*-axis.

The distance from the inferior margin of the symphysis pubis to the bladder neck (SPBN) and dorsocaudal linear movement (DLM) measurements were used to quantify the mobility of the bladder neck. The SPBN assessed the vertical movement of the bladder neck, and was obtained by measuring the distance represented on the *X*-axis between the bladder neck and the pubic symphysis. DLM was used to evaluate the horizontal displacement of the bladder neck toward the posterior, and was obtained by measuring the distance represented on the Y axis between the pubic symphysis and bladder neck.

**Figure 1:**
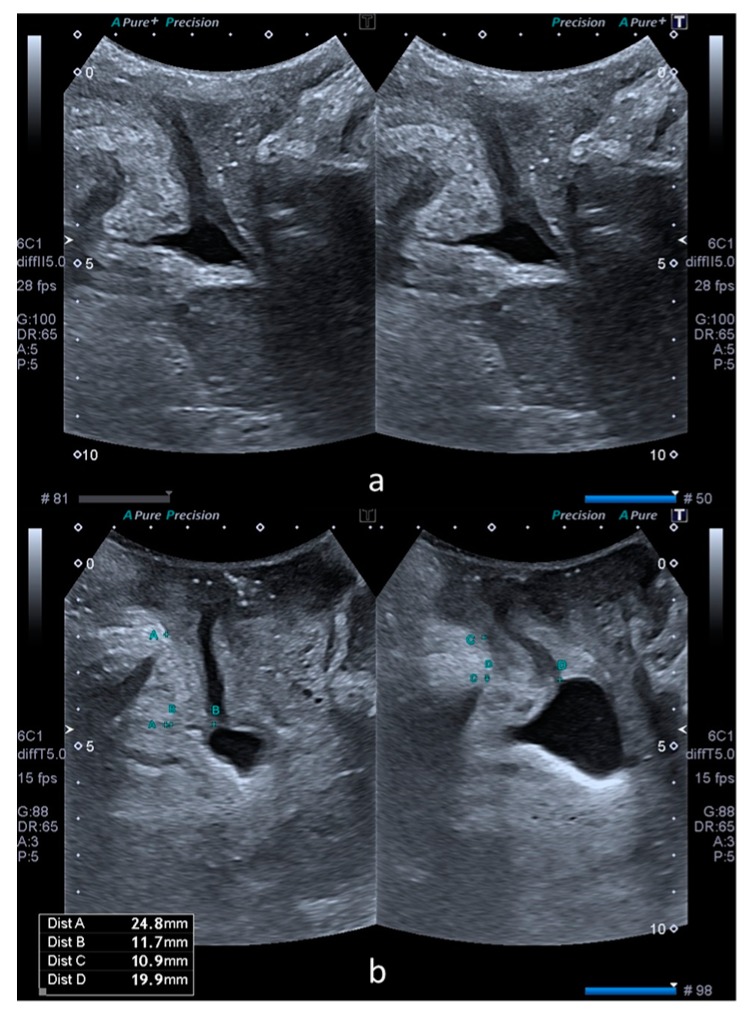
Comparative transperineal US images at rest (left) and on Valsalva maneuver (right). Image (**a**) was obtained from a patient without a cystocele. Image (**b**) was obtained from a patient with a cystocele. Image b also shows the measurements performed in the XY coordinate system. Dist A (left) and dist C (right) correspond to SPBN and dist B (left) and dist D (right) correspond to DLM.

The bladder neck mobility was quantified by subtracting SPBN and DLM values at rest and on Valsalva, with the results being noted as ΔSPBN and ΔDLM. Subsequently, the rest, Valsalva and subtraction values of SPBN and DLM were compared between the cystocele and control groups. Figure 1 illustrates these parameters in a patient from the control group (a) and another patient from the case group (b).

The potential correlations of the number of vaginal births and the heaviest newborn’s birth weight with SPBN and DLM were also investigated. The correlation analysis was performed separately for the following groups: SUI without a cystocele, SUI with a cystocele, and SUI regardless of the presence of a cystocele (the previous two groups pooled together).

GraphPad Prism 8 was used for statistical analysis. The Mann–Whitney test was used to analyze the difference between medians where numerical variables were compared. The Spearman coefficient was used to measure the correlation between the variables. The results were considered significant when the *p*-value was less than 0.05.

## 3. Results

According to the baseline patient’s characteristics presented in Table 2, no statistically significant differences were documented between the groups (*p* > 0.05).

**Figure 2:**
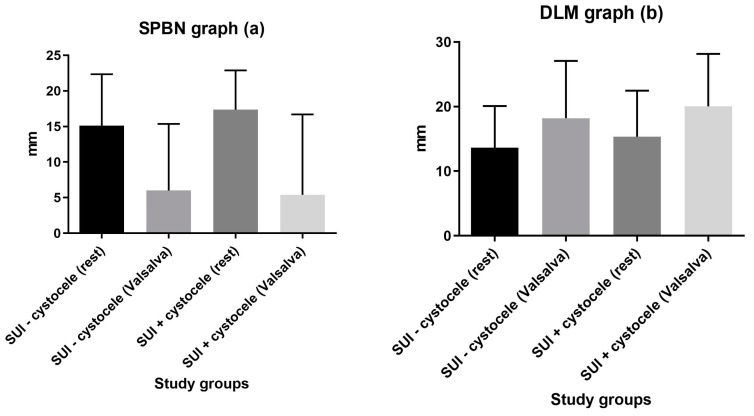
Groups comparison of SPBN (**a**) and DLM (**b**) values at rest vs. on Valsalva.

The SPBN values were significantly higher at rest than on Valsalva for both control (15.38 ± 7.21 mm vs. 6.48 ± 9.42 mm; *p* < 0.0001) and cystocele (17.28 ± 6 mm. vs. 5.36 ± 8 mm; *p* < 0.0001) groups. The DLM values were significantly lower at rest than at Valsalva for both control (13.47 ± 6.37 mm vs. 18.22 ± 8.8 mm; *p* = 0.0002) and cystocele (15.08 ± 7.16 mm vs. 20.04 ± 8.11 mm; *p* = 0.0014) groups (Figure 2).

There was no significant difference between the two groups regarding SPBN and DLM at rest or on Valsalva (Table 3). The bladder neck mobility as measured using ΔSPBN was higher in the cystocele group (9.1 ± 6.3 mm vs. 11.9 ± 10.9 mm), but the difference was not statistically significant (*p* = 0.2). ΔDLM had similar values in the control group (4.7 ± 5.3 mm) and cystocele group (4.9 ± 8.1 mm), with no significant difference (*p* = 0.6).

The bladder neck mobility measurements had no significant correlation with either the number of vaginal births or the heaviest newborn’s weight, for both the SUI without cystocele group and the SUI with cystocele group. A tendency toward statistical significance was noted for the ΔSPBN—heaviest newborn’s birth weight correlation in the SUI without cystocele group (*p* = 0.07), and for the ΔDLM—number of vaginal births correlation, also in the SUI without cystocele group (*p* = 0.06). When the groups were pooled together, a significant positive correlation was found between ΔSPBN and the heaviest newborn’s birth weight (*p* = 0.04) in women with SUI, regardless of the presence of a cystocele. No significant correlations between ΔSPBN and parity, or between ΔDLM and the heaviest newborn’s birth weight or number of vaginal births were found in the pooled group. The correlations are presented in Table 4.

## 4. Discussion

Bladder neck’s position and mobility can be assessed by transperineal US with a high degree of reliability [15]. Measurements of the bladder neck position were performed at rest and on maximal Valsalva maneuver, and the differences yielded numerical values for bladder neck displacement. On Valsalva, the proximal urethra may be displaced in a posteroinferior direction. There is “no specific definition of normal” for bladder neck displacement, although cut-offs between 15–25 mm have been proposed to define hypermobility [16,17].

In order to achieve higher accuracy for the assessment of the bladder neck mobility, we assessed bidirectional rather than unidirectional parameters, in an XY-coordinate system. This study showed that while the average bladder neck displacement, as measured by ΔSPBN, was slightly higher in the cystocele group, the difference from the control group was not statistically significant. In addition, ΔDLM had similar values in the control and cystocele group. Our results are in line with Meyer’s et al. study [18], who evaluated the effects of different factors on bladder neck position and mobility, in nulliparous and parous women, using transperineal US. These factors were: Spontaneous and instrumented deliveries, the baby’s birthweight, the presence of SUI, and the woman’s age and weight. Meyer et al. demonstrated that the extent of bladder neck displacement was not significantly different between the groups based on parity, except in SUI patients, where a significantly more mobile bladder neck was seen as compared to continent women [18].

When analyzed separately, the groups yielded no significant correlation between the bladder neck mobility measurements and the newborn’s weight/number of vaginal births. However, the tendency toward significance for the ΔSPBN—heaviest newborn’s birth weight and ΔDLM—number of vaginal births correlations in the SUI without cystocele group is worth mentioning. This shows that both the heaviest newborn’s birth weight and number of vaginal births might have an impact on the mobility of the bladder neck in the SUI without cystocele group. In this case, both r values showed a positive correlation of 0.3. When the two groups were pooled together, the ΔSPBN—heaviest newborn’s birth weight correlation was statistically significant, suggesting that the lack of significance in the SUI with cystocele group might have been caused by the lower sample size.

With regards to the newborn’s weight, our study found a significant positive correlation between ΔSPBN and the heaviest newborn’s birth weight in women with SUI, regardless of the presence of a cystocele. The clinical impact of this observation is that the heaviest newborn’s birth weight may be considered a risk factor for bladder neck hypermobility, leading to SUI. In contrast, Meyer et al. reported no correlation between bladder neck displacement and newborns’ birthweight, but the presence of a cystocele was not documented [18]. However, our study is in line with longstanding evidence [19,20] that indicates that high birth weight may lead to bladder neck hypermobility. A reason for this phenomenon may be pudendal nerve damage that occurs at birth, [19,20] leading to a lack of strength of perineal tissues and urethral hypermobility.

The other potential risk factor for bladder neck hypermobility, DLM, did not show any significant correlation neither with parity nor with the heaviest newborn’s birth weight.

One limitation of this study was the small number of patients in each study group. A second limitation is related to the transperineal US technique, and refers to possible confounders such as urinary bladder filling and levator ani co-activation. Both factors are likely to influence the bladder neck displacement, which is reflected by the heterogeneity of the bladder neck mobility values reported in the literature [18,19,20]. As it has been demonstrated that the bladder neck is less mobile when it is full [8], we examined the patients in a 30–45 min post voiding interval in order to avoid mobility limitation. However, we could not find any way to avoid levator ani co-activation, and this fact represents another limitation of this study. Due to the above-mentioned reasons, the difficulty of comparing our findings with other outcomes published in the literature and the necessity of standardized measurement protocols arise. A standardized approach in terms of the transperineal US conditions (for example, the degree of bladder filing when the measurements are performed) could increase the reproducibility of the results in future research. Despite these limitations, our study’s findings contribute to a better understanding of the bidimensional bladder neck mobility in cases where SUI and a cystocele coexist, and it might strengthen the evidence on which the counseling of patients is based.

## 5. Conclusions

The purpose of this study was to assess the influence of a cystocele on the mobility of the bladder neck in women with stress urinary incontinence (SUI) using a transperineal US technique. The presence of a cystocele had no significant impact on the bladder neck mobility measurements in patients with SUI. There was no significant correlation between the bladder neck mobility measurements and the newborn’s weight/number of vaginal births, when the SUI without cystocele and the SUI with cystocele groups were analyzed separately. However, the heaviest newborn’s birth weight positively correlated with the mobility of the bladder neck as quantified by SPBN, when the groups were analyzed together.

## Figures and Tables

**Table 1:** Inclusion and exclusion criteria for a transperineal ultrasound (US) study of patients with stress urinary incontinence (SUI), with or without an associated cystocele.

Inclusion Criteria	Exclusion Criteria
**Women with SUI** **• with grade 2 or 3 cystocele** **• without cystocele**	**Women who had history of vaginal surgery** **• for SUI or** **• for pelvic organ prolapse**
**Women who had ≥1 vaginal birth**	**Nulliparous women**
**Women who delivered exclusively vaginally**	

**Table 2:** Study population characteristics. (SD = standard deviation).

	SUI without Cystocele (Mean, SD)	SUI with Cystocele (Mean, SD)	*p* Value
**Number of patients**	33	38	
**Age (years old)**	57.9 ± 8.8	59.9 ± 9.3	0.3
**BMI (kg/m^2^)**	28.3 ± 5.4	29 ± 5.1	0.4
**Number of vaginal births**	2.6 ± 1.0	2.4 ± 1.4	0.2
**Heaviest newborn’s birth weight (g)**	3569 ± 206.7	3513 ± 473.7	0.6

**Table 3:** Parameters describing bladder neck mobility.

	SUI without Cystocele (Mean, SD)	SUI with Cystocele (Mean, SD)	*p* Value
SPBN Rest (mm)	15.1 ± 7.2	17.3 ± 5.5	0.1
SPBN Valsalva (mm)	6.0 ± 9.3	5.4 ± 11.3	0.8
ΔSPBN (mm)	9.1 ± 6.3	11.9 ± 10.9	0.2
DLM Rest (mm)	13.6 ± 6.4	15.3 ± 7.1	0.4
DLM Valsalva (mm)	18.2 ± 8.8	20.0 ± 8.1	0.3
ΔDLM (mm)	4.7 ± 5.3	4.9 ± 8.1	0.6

**Table 4:** Spearman correlation coefficients showing the correlations between ΔSPBN/ΔDLM and the number of vaginal births/heaviest newborn’s birth weight in women with SUI. The correlations are shown separately for patients with SUI alone, SUI and a cystocele, and for both groups pooled together.

		Spearman r Correlation	95% CI	*p* Value
ΔSPBN—Number of vaginal births	SUI without cystocele	0.2	0.1–0.6	0.2
	SUI with cystocele	0.04	0.3–0.4	0.8
	Combined	0.09	0.2–0.3	0.5
ΔSPBN—Heaviest newborn’s birth weight	SUI without cystocele	0.3	0.05–0.7	0.07
	SUI with cystocele	0.2	0.1–0.5	0.1
	Combined	0.2	0.001–0.5	0.04
ΔDLM—Number of vaginal births	SUI without cystocele	0.3	0.04–0.7	0.06
	SUI with cystocele	−0.008	0.2–0.3	0.9
	Combined	0.1	0.1–0.4	0.4
ΔDLM—heaviest newborn’s birth weight	SUI without cystocele	0.2	0.1–0.6	0.1
	SUI with cystocele	−0.2	0.05–0.1	0.1
	Combined	0.08	0.1–0.3	0.5

## Supplementary Materials

The following are available online at https://www.mdpi.com/1010-660X/55/9/562/s1, Table S1. Inclusion and exclusion criteria for a transperineal US study of patients with SUI, with or without an associated cystocele, Figure S1. Comparative transperineal US images at rest (left) and on Valsalva maneuver (right). Image a was obtained from a patient without cystocele. Image b was obtained from a patient with cystocele. Image b also shows the measurements performed in the XY coordinate system. Dist A (left) and dist C (right) correspond to SPBN and dist B (left) and dist D (right) correspond to DLM, Table S2: Study population characteristics. (SD = standard deviation), Figure S2. Groups comparison of SPBN and DLM values at rest vs. on Valsalva, Table S3. Parameters describing bladder neck mobility, Table S4. Spearman correlation coefficients showing the relations between ΔSPBN/ΔDLM and number of vaginal births/heaviest newborn’s birth weight. The correlations are shown separately for patients with SUI alone, SUI and cystocele and for both groups pooled together.
