# Supplementary material for: Transperineal Ultrasound Assessment of a Cystocele’s Impact on the Bladder Neck Mobility in Women with Stress Urinary Incontinence
Source: Medicina (Kaunas). 2019 Sep 3;55(9):562. doi: 10.3390/medicina55090562 (PMC6780490; doi:10.3390/medicina55090562)
Supplement: Supplementary file 1 [file medicina-55-00562-s001.zip › Anexa 1. Formular_Consimtamant_Informat PMFIUF-1.doc]

**FORMULAR DE CONSIMȚĂMÂNT INFORMAT**

*□ Sunt un participant adut în acest studiu*

***Numele participantului****____________________________*

**CNP** **	**Telefon** ____________________

*□ Sunt părintele sau tutorele ce oferă consimțământul informat în numele participantului*

*Numele participantului____________________________*

**Telefon** ____________________

**Participați la alte studii clinice**? □ Da	□ Nu

**OBIECTIVUL CERCETĂRII/ STUDIULUI**

Sunteți invitată să participați la un studiu de cercetare al incontinenței urinare. Ne propunem standardizarea diagnosticului acestei patologii. Ați fost selectată ca un posibil participant la acest studiu, deoarece ați fost diagnosticată cu incontinența urinară.

**PARTICIPARE VOLUNTARĂ**

Participarea dumneavoastră este cu totul voluntară. Decizia de a participa vă aparține și veți putea să vă retrageți oricând din acest studiu.

Decizia dumneavoastră de a nu participa nu va avea nici un efect negativ asupra dumneavoastră sau asupra îngrijirii medicale pe care o primiți.

Dacă decideți să vă retrageți din acest studiu, vă rugăm să o notificați pe Dr. Maria-Patricia Rada la numărul de telefon 0740378450 sau adresa de e-mail Rada.Maria@umfcluj.ro

**PROCEDURI**

Dacă decideți să participați, investigatorul principal, Dr. Rada Maria și echipa de cercetare vor efectua analize de laborator, examinarea ecografica a planșeului pelvin și, eventual, explorări urodinamice pentru pacientele înrolate în studiu.

Următoarele proceduri vor putea fi efectuate în cadrul studiului:

- Recoltare de urină / de sânge;
- Determinări bacteriologice ale probelor recoltate;
- Examinarea ecografică a planșeului pelvin (abord transperineal) care poate presupune și instilarea unor substanțe de contrast la nivelul vezicii urinare;
- Examinări urodinamice (care presupun utilizarea unor catetere inserate la nivelul vezicii urinare precum și instilarea serului fiziologic la nivelul vezicii urinare);

Toate procedurile utilizate sunt în conformitate cu protocoalele diagnostice existente la ora actuală.

Probele recoltate vor fi prelucrate / analizate în cadrul Clinicii Obstetrică-Ginecologie ˮDominic Stancaˮ, precum și în laboratoare din afara acestei instituții care dețin echipamentul necesar pentru determinările din cadrul studiului.

Explorările ecografice se vor desfășura în cadrul Clincii Obstetrică-Ginecologie II, ˮDominic Stancaˮ, Cluj-Napoca, fiind posibilă înrolarea în studiu a unor paciente examinate în alte centre. Explorările urodinamice se vor desfășura într-un centru care dispune de echipamentul necesar acestor evaluări.

Probele de urină recoltate vor fi păstrate la o temperatură de -20°C până la prelucrare iar cele sanguine vor fi centrifugate în vederea obținerii serului în 30 minute de la recoltare; acesta va fi stocat la -40°C până la prelucrare.

Probele rămase la finalul studiului nu vor fi păstrate pentru cercetări viitoare.

Probele din studiu nu vor fi utilizate pentru testare genetică.

**DURATA STUDIULUI**

Prezenta cercetare va dura 4 ani și implică participarea dvs. activă in intervalul 2017-2021 și colectarea de informații medicale pe parcursul întregului interval.

**RISCURI**

Pot exista riscuri, disconfort și neplăceri asociate cu orice studiu de cercetare. Acestea merită o analiză atentă.

Riscurile asociate cu instilarea intravezicală a unor substanțe și cu testările urodinamice sunt:

- iritație temporară la nivelul uretrei
- senzația imperioasă de a urina
- disconfort abdominal
- infecții urinare
- procedurile pot implica și riscuri imprevizibile în prezent

**BENEFICII**

Participarea dumneavoastră ne va ajuta să găsim răspunsul la întrebările din această cercetare și beneficiul dumneavoastra este reprezentat de posibilitatea obținerii unui diagnostic acurat al tipului de incontinență urinară de care suferiți, în mod gratuit. În cazul în care ca urmare a acestui studiu se va implementa un algoritm complet de diagnostic al incontinenței urinare, întreaga comunitate care suferă de aceasta patologie va putea beneficia de diagnostic si consiliere optimă în vederea reușitei tratamentului.

**RESPONSABILITĂȚILE PARTICIPANTULUI**

Ca participant la acest studiu, responsabilitățile dvs. includ:

- Să urmați instrucțiunile/recomandările ce vă sunt oferite de echipa de cercetare.
- Să completați calendarul micțional și chestionarele aferente studiului conform recomandărilor medicului.
- Să vă prezentați la vizitele de studiu, conform programărilor. În cazul în care nu vă puteți prezenta, vă rugăm să o informați pe Dr. Rada Maria-Patricia la numărul de telefon 0740378450.
- Să prezentați documentele medicale solicitate la fiecare vizită medicală.
- Să anunțați investigatorul dacă urmați un tratament la momentul înrolării în studiu / desfașurarii procedurilor din cadrul studiului, daca prezentați efecte adverse sau aveți alte spitalizări sau vizite la medic pe perioada desfășurării studiului, sunteți însărcinată.
- Să adresați întrebări atunci când considerați necesar.

**DREPTURILE PARTICIPANTULUI**

- Nu trebuie să vă simțiți obligat să participați la acest studiu. Trebuie să vă acordați suficient timp pentru a lua o decizie.
- Întrebările dumneavoastră trebuie să primească un răspuns clar și satisfăcător.
- În cazul în care vă decideți să nu mai participați, puteți oricând transmite investigatorului decizia dumneavoastră.
- Orice informații noi, importante, aflate în cursul desfășurării acestui studiu de cercetare, care ar putea afecta starea dumneavoastră de sănătate sau dorința dumneavoastră de a continua participarea la acest studiu, vă vor fi aduse la cunoștință în timp util.

**RETRAGEREA DIN STUDIU**

În cazul în care sunteți inițial de acord să participați la acest studiu și apoi vă răzgândiți, sunteți liberi să vă retrageți consimțământul și să întrerupeți participarea la studiu în orice moment. Decizia dumneavoastră nu va afecta dreptul dvs. de a primi îngrijiri medicale pentru bolile de care suferiți și nu veți pierde nici un beneficiu la care altfel ați fi îndreptățită.

Dacă decideți să vă retrageți din acest studiu, vă rugăm să o notificați pe Dr. Rada Maria-Patricia la numărul de telefon 0740378450.

Dacă vă retrageți din studiu nu va exista niciun fel de urmare.

Investigatorul poate, de asemenea, să vă retragă din studiu fără consimțământul dumneavoastră, pentru unul sau mai multe dintre următoarele motive:

- Nerespectarea instrucțiunilor investigatorului și a personalului din studiu.
- Investigatorul decide că o continuare a participării ar putea fi dăunătoare pentru dvs.
- Pe durata desfășurării studiului aveți nevoie de un tratament asociat, ce influențează acest studiu.
- Studiul este anulat.
- Alte motive administrative.
- Circumstanțe neprevăzute.

**CONFIDENȚIALITATE**

Rezultatele acestui studiu de cercetare pot fi prezentate la manifestări științifice sau medicale sau publicate în reviste științifice. Identitatea și /sau informațiile dumneavoastră personale nu vor fi divulgate, cu excepția celor autorizate de dvs. sau în conformitate cu legea. Numai cercetătorii vor avea acces la datele/identitatea dvs. și aceste informații vor fi protejate într-un loc cu acces restricționat. Aceste informații nu vor fi dezvăluite nimănui cu excepția echipei implicate în această cercetare conform legislației actuale. Cu toate acestea, există întotdeauna riscul ca informația, chiar dacă este atent protejată, să poată fi identificată.

**CONSIDERAȚII FINANCIARE**

Nu veți fi plătit pentru participarea la acest studiu.

Nu există nici un cost suplimentar pentru dvs. pentru a participa la acest studiu, altele decât cheltuielile de bază, cum ar fi transportul și timpul personal, necesare pentru a se prezenta la toate vizitele incluse în studiu.

**ORGANIZAREA ȘI FINANȚAREA STUDIULUI**

Acest studiu se va desfășura în cadrul unui proiect de doctorat.

**INFORMAȚII DE CONTACT**

Dacă aveți întrebări, nelămuriri sau reclamații cu privire la acest studiu de cercetare, procedurile, riscurile și beneficiile, puteți să vă adresați investigatorului Dr. Rada Maria-Patricia.

Telefon: 0740378450

E-mail: Rada.Maria@umfcluj.ro

**Semnând mai jos, înseamnă că sunteți de acord să participați în acest studiu și confirmați faptul că ați avut posibilitatea de a pune întrebări și că vi s-a oferit un răspuns satisfăcător, că ați înțeles scopul studiului, procedurile și riscurile pe care acesta le presupune.**

**Veți primi o copie a acestui formular de consimțământ semnat și datat.**

_____________________________________	____________
Semnătura Participantului	Data

_____________________________________

Numele Participantului

Dacă este cazul:

_____________________________________	____________

Semnătura Reprezentantului Legal	Data

_____________________________________

Numele Reprezentantului Legal

_____________________________________

Autoritatea Reprezentantului Legal de a semna pentru Participant

(de exemplu, părinte, tutore, etc)

____________________________________	___________
Semnătura Persoanei ce obține consimțământ	Data

_____________________________________

Numele Persoanei ce obține consimțământul
