# Supplementary material for: Transperineal Ultrasound Assessment of a Cystocele’s Impact on the Bladder Neck Mobility in Women with Stress Urinary Incontinence
Source: Medicina (Kaunas). 2019 Sep 3;55(9):562. doi: 10.3390/medicina55090562 (PMC6780490; doi:10.3390/medicina55090562)
Supplement: Supplementary file 1 [file medicina-55-00562-s001.zip › Informed consent - En translation.doc]

**INFOREMD CONSENT FORM**

*□ I am an adult person participating to this study*

***Name of the participant****____________________________*

**National personal number** **	**Phone nr** ____________________

*□ I am the parent or tutor of the patient, offering informed consent on his behalf*

***Name of the participant*** *____________________________*

**Phone nr** ____________________

**Do you participate to other clinical studies**? □ Yes	□ N

**STUDY OBJECTIVES**

You are invited to participate in a urinary incontinence research study. We aim to standardize the diagnosis of this pathology. You have been selected as a possible participant in this study because you have been diagnosed with urinary incontinence

**PARTICIPARE VOLUNTARĂ**

Your participation is entirely voluntary. The decision to participate belongs to you and you will be able to withdraw from this study at any time.

Your decision not to participate will have no adverse effect on you or the medical care you receive.

If you decide to withdraw from this study, please notify Dr. Maria-Patricia Rada at phone number 0740378450 or email [Rada.Maria@umfcluj.ro](mailto:Rada.Maria@umfcluj.ro)

**PROCEDURES**

If you decide to participate, the principal investigator, Dr. Rada Maria and the research team will carry out laboratory analyzes, ultrasound examination of the pelvic floor and, possibly, urodynamic examinations for the patients enrolled in the study. The following procedures may be performed within the study:

• Urine / blood collection;

• Bacteriological determinations of the collected samples;

• Ultrasound examination of the pelvic floor (transperineal approach) which may also involve the instillation of contrast agents in the bladder;

• Urodynamic examinations (which involve the use of catheters inserted in the bladder as well as the instillation of the physiological serum in the bladder);

All the procedures used are in accordance with the current diagnostic protocols.

The collected samples will be processed / analyzed in the Obstetrics-Gynecology Clinic ˮDominic Stancaˮ, as well as in laboratories outside this institution that have the necessary equipment for the determinations in the study.

The ultrasound examinations will be carried out within the Obstetrics-Gynecology II Clinic, „Dominic Stancaˮ, Cluj-Napoca, and it is possible to enroll patients examined in other centers. The urodynamic explorations will be carried out in a center that has the necessary equipment for these evaluations.

The collected urine samples will be kept at a temperature of -20 ° C until processing and the blood samples will be centrifuged in order to obtain the serum within 30 minutes from the collection; it will be stored at -40 ° C until processing.

The remaining samples at the end of the study will not be kept for future research.

The samples in the study will not be used for genetic testing.

**STUDY DURATION**

This research will last 4 years and involves your active participation in the period 2017-2021 and the collection of medical information throughout the whole period.

**RISKS**

There may be risks, discomfort and inconvenience associated with any research study. They deserve careful consideration.

The risks associated with the intravesical instillation of some substances and the urodynamic tests are:

• temporary irritation of the urethra

• the urge to urinate

• abdominal discomfort

• urinary tract infections

• the procedures may also involve unpredictable risks at present

**BENEFITS**

Your participation will help us find the answers to the questions in this research and your benefit is the possibility of obtaining an accurate diagnosis of the type of urinary incontinence you are suffering, for free. If a complete algorithm for diagnosing urinary incontinence will be implemented as a result of this study, the entire community suffering from this pathology will be able to benefit from optimal diagnosis and counseling in order to be successful in the treatment.

**RESPONSIBILITIES OF THE PARTICIPANT**

As a participant in this study, your responsibilities include:

• Follow the instructions / recommendations provided by the research team.

• Complete the micturition calendar and the study questionnaires according to the doctor's recommendations.

• To attend the study visits, as scheduled. In case you cannot attend, please inform Dr. Rada Maria-Patricia on the phone number 0740378450.

• Present the required medical documents at each medical visit.

• To notify the investigator if you are undergoing treatment when enrolling in the study / conducting the procedures in the study, if you have any adverse effects or have other hospitalizations or doctor visits during the course of the study, you are pregnant.

• Ask questions when you deem it necessary.

**PARTICIPANT`S RIGHTS**

• You do not have to feel obligated to participate in this study. You have to give yourself enough time to make a decision.

• Your questions should receive a clear and satisfactory answer.

• If you decide to stop participating, you can always send your decision to the investigator.

• Any important new information during the course of this research study, which could affect your health or your desire to continue participating in this study, will be made known to you in a timely manner.

**RETREATING FROM THE STUDY**

If you initially agree to participate in this study and then change your mind, you are free to withdraw your consent and discontinue participating in the study at any time. Your decision will not affect your right to receive medical care for the diseases you suffer and you will not lose any benefit you would otherwise be entitled to.

If you decide to withdraw from this study, please notify Dr. Rada Maria-Patricia at telephone number 0740378450.

If you withdraw from the study there will be no consequence.

The investigator may also withdraw from the study without your consent, for one or more of the following reasons:

• Failure to follow the instructions of the investigator and the study personnel.

• The investigator decides that further participation may be harmful to you.

• During the course of the study you need an associated treatment, which influences this study.

• The study is canceled.

• Other administrative reasons.

• Unforeseen circumstances.

**CONFIDENTIALITY**

The results of this research study can be presented at scientific or medical events or published in scientific journals. Your identity and / or personal information will not be disclosed, except as authorized by you or in accordance with the law. Only researchers will have access to your data / identity and this information will be protected in a restricted place. This information will not be disclosed to anyone except the team involved in this research under current legislation. However, there is always a risk that the information, even if carefully protected, may be identified.

**FINACIAL CONSIDERATIONS**

You will not be paid for participating in this study.

There is no additional cost for you to participate in this study, other than basic expenses, such as transportation and personal time, required to attend all visits included in the study.

**ORGANIZATION AND FUNDING OF THE STUDY**

This study will be carried out within a doctoral project.

**CONTACT INFORMATION**

If you have questions, concerns or complaints regarding this research study, procedures, risks and benefits, you can contact the investigator Dr. Rada Maria-Patricia.

Phone: 0740378450

E-mail: [Rada.Maria@umfcluj.ro](mailto:Rada.Maria@umfcluj.ro)

**By signing below, you agree to participate in this study and confirm that you have had the opportunity to ask questions and that you have been given a satisfactory answer, that you have understood the purpose of the study, the procedures and the risks involved.**

**You will receive a copy of this signed and dated consent form.**

_____________________________________	____________

Signature of Participant	Date

_____________________________________

Name of the Participant

If appropriate:

_____________________________________	____________

Signature of Legal Representative	Date

_____________________________________

Name of Legal Representative

_____________________________________

Authority of the Legal Representative to sign for the Participant

(eg parent, guardian, etc.)

____________________________________	___________

Signature of the person obtaining consent	Date

_____________________________________

The name of the person obtaining the consent
